# Recurrence of Non-Melanoma Skin Cancers in the Head and Neck Area—A Single-Center Retrospective Analysis

**DOI:** 10.3390/jcm15114196

**Published:** 2026-05-29

**Authors:** Monika Wojarska, Karol Mitas, Paulina Bernecka, Maria Gac, Amelia Maria Glinko, Samira Kierat, Gabriela Ratajczyk, Marija Turek, Adrianna Włoch, Krzysztof Pastuszak, Jerzy Jankau

**Affiliations:** 1 Student Scientific Circle of Plastic Surgery, Faculty of Medicine, Medical University of Gdańsk, 80-210 Gdańsk, Poland; monika.wojarska@gumed.edu.pl (M.W.); k.mitas@gumed.edu.pl (K.M.); mariagac@gumed.edu.pl (M.G.); amelia.glinko@gumed.edu.pl (A.M.G.); kieratsamira@gumed.edu.pl (S.K.); gabrysia.ratajczyk@gumed.edu.pl (G.R.); m.turek@gumed.edu.pl (M.T.); adrianna.wloch@gumed.edu.pl (A.W.); jerzy.jankau@gumed.edu.pl (J.J.); 2 Plastic Surgery Department, Medical University of Gdańsk, 80-210 Gdańsk, Poland; 3 Department of Algorithms and Systems Modelling, Faculty of Electronics, Telecommunications and Informatics, Gdańsk University of Technology, 80-233 Gdańsk, Poland; krzpastu@pg.edu.pl; 4 Laboratory of Translational Oncology, Intercollegiate Faculty of Biotechnology of the University of Gdańsk and Medical University of Gdańsk, 80-210 Gdańsk, Poland; 5 Centre of Biostatistics and Bioinformatics, Medical University of Gdańsk, 80-210 Gdańsk, Poland

**Keywords:** non-melanoma skin cancer, local recurrence, head and neck tumors, basal cell carcinoma, risk factors

## Abstract

**Background**: Non-melanoma skin cancers (NMSCs) of the head and neck represent a therapeutic challenge due to the region’s complex anatomy, functional considerations, and frequent involvement of high-risk anatomical zones. Local recurrence remains a clinically significant concern, however real-world data regarding recurrence patterns and associated risk factors in facial NMSCs are limited. **Objectives**: To evaluate the incidence of local recurrence of facial skin cancers after surgical treatment and to determine clinicopathological and anatomical actors associated with an increased risk of recurrence. **Methods**: In this single-center retrospective cohort study, consecutive patients undergoing surgical excision of facial NMSC were included. The treatment of choice was always surgical excision under general or local anesthesia, with an adequate margin of macroscopically unchanged tissue. Mohs surgery was not used, and none of the patients received immunosuppression. Clinical and pathological data were extracted from medical records. Histopathological examination constituted the basis for establishing the final clinical diagnosis and thus was not verified otherwise. The primary outcome was histologically confirmed local recurrence defined as the reappearance of a tumor of the same histopathological type at the same anatomical site as the previously excised lesion. Patients in the non-recurrence group were defined as those who did not experience any recurrence within a 5-year follow-up period after the initial surgical treatment. Fisher’s exact test and the Mann–Whitney U test were used for statistical analysis. Logistic regression was performed to explore factors associated with recurrence. Due to incomplete follow-up data for the non-recurrent group, we limited the timing analysis to recurrent cases only, as these limitations precluded the use of standard survival analysis. Results: A total of 302 lesions were analyzed, with recurrence status available for 291 tumors. The overall recurrence rate was 28.52%. Basal cell carcinoma (BCC) was the most common histopathological subtype. Recurrences occurred more frequently in anatomically high-risk areas, particularly the scalp, temple and nose. Infiltrative BCC subtypes demonstrated higher recurrence rates than nodular and superficial subtypes. Patients with recurrent tumors were younger than those without recurrence. A history of prior skin radiotherapy was associated with increased odds of recurrence. Tumor size and surgical margin width were not significantly associated with recurrence. Multivariate models showed limited discriminatory ability, suggesting that additional unmeasured factors contribute to recurrence risk. **Conclusions**: Local recurrence of non-melanoma skin cancers in the head and neck region remains a substantial clinical concern, particularly in high-risk anatomical sites and tumors with aggressive histopathological features. These findings highlight the importance of long-term follow-up and support further prospective studies to improve recurrence risk assessment and treatment strategies.

## 1. Introduction

Non-melanoma skin cancers (NMSC) represent the most common malignancies worldwide [1]. Over recent decades, a continuous increase in their incidence has been observed [2,3,4,5], which is attributed to prolonged life expectancy and increasing exposure to ultraviolet (UV) radiation [6,7].

The main subtypes of non-melanoma skin cancers are basal cell carcinoma (BCC), accounting for approximately 80% of cases, and squamous cell carcinoma (SCC), representing about 20%. Together, these entities constitute nearly 99% of all NMSC cases [8]. Although they rarely lead to distant metastases or death, if left untreated they may infiltrate and destroy adjacent tissues [9,10,11]. Exposure to UV radiation is the principal risk factor for the development of NMSC, which explains their predominant localization in sun-exposed areas such as the face, neck, and extremities [8,9]. Due to the frequent involvement of the head and neck region, these tumors significantly affect not only patients’ health but also their quality of life, aesthetic appearance, and social functioning.

Surgical excision with histologically clear margins remains the gold standard in the treatment of NMSC [10]. A surgical margin of 2–4 mm is typically considered adequate to ensure complete tumor removal. However, surgical management of facial skin cancers often carries a risk of permanent disfigurement and functional impairment of critical anatomical structures, including the eyelids, oral commissure, or nose. Consequently, additional reconstructive procedures are frequently required to restore function and improve patients’ quality of life. However, over the past few years minimally invasive methods, such as electrochemotherapy (ECT) and topical chemotherapy, have gained increasing attention. Electrochemotherapy has been reported as effective, especially in selected cases of NMSC [12]. Local chemotherapy with use of 5-fluorouracil demonstrates efficacy mainly in treating superficial BCC and SCC in situ [13]. Systematic reviews suggest comparable short-term effectiveness in carefully selected superficial lesions, whereas its effectiveness is limited in deeper tumors, which are associated with higher risk of recurrence [12,13].

Despite surgical treatment, recurrence rates for BCC range from 0.3% to 6.5% [11], while those for SCC vary between 5% and 20% [14]. Determining the precise recurrence rate of NMSC remains challenging due to the lack of a standardized patient follow-up protocol and unified criteria for defining recurrence. The risk of recurrence is influenced by multiple factors, including the surgical technique used, history of prior recurrence, tumor size and anatomical location, depth of invasion, and histopathological grade [2,9,15,16].

Previous studies on recurrent NMSC have predominantly evaluated lesions occurring across the entire body [11,14]. In contrast, analyses specifically addressing recurrence rates in the facial region remain limited. Lesions located in the head and neck area, particularly within the so-called H-zone, are classified as high-risk skin cancers, due to their increased propensity for deep invasion, recurrence, and, in selected cases, metastasis [17]. The H-zone includes the nose, eyelids, eyebrows, periorbital region, ears, preauricular area, and central face, and is defined by the proximity of critical anatomical structures as well as considerable variability in skin thickness across different subregions [18].

The aim of the present study was to evaluate the recurrence rate of skin cancers of the head and neck in patients treated surgically at the Department of Plastic Surgery, University Clinical Center, between 2020 and 2024.

## 2. Materials and Methods

This retrospective cohort study analyzed clinical and pathological factors associated with non-melanoma skin cancer recurrence following surgical excision at a single institution. A study was conducted of patients hospitalized for head and neck skin cancers who underwent surgical treatment at the Department of Plastic Surgery, University Clinical Center, between 2020 and 2024. Histopathological data, including final diagnosis and information about completeness of excision and/or margins were obtained from original pathology reports. No independent re-evaluation of the pathology findings was performed for the purposes of this study.

A total of 302 lesions were included in the analysis. The primary outcome was histologically confirmed local recurrence defined as the reappearance of a tumor of the same histopathological type at the same anatomical site as the previously excised lesion, with 291 lesions having known outcome status. Patients classified as non-recurrent were those without evidence of recurrence during a minimum 5-year follow-up period.

Categorical variables were summarized as frequencies and percentages; continuous variables as median with interquartile range (IQR). Group comparisons used Fisher’s exact test for categorical variables (with Cramér’s V as effect size measure) and Mann–Whitney U test for continuous variables (with rank-biserial correlation as effect size). Logistic regression estimated odds ratios (OR) with 95% confidence intervals (CI). Time to documented recurrence among recurrent lesions was visualized as the cumulative distribution of months from surgery to recurrence. Considering the lack of complete follow-up data for non-recurrent lesions, we could not perform a standard Kaplan–Meier recurrence-free survival analysis. Instead, we focused on the timing of relapses among recurrent cases only. These data should not be misinterpreted as survival estimates for the entire cohort.

Multiple lesions per patient may exist in this dataset. Standard statistical tests assume independence between observations; therefore, standard errors may be underestimated and *p*-values should be interpreted cautiously. All analyses were performed using R version 4.3.2 (31 October 2023).

## 3. Results

A total of 302 lesions were included in the analysis, with complete recurrence data available for 291 lesions. Local recurrence was identified in 83 cases, corresponding to an overall recurrence rate of 28.52%.

Median patient age was 73 years (IQR 66–81; range 33–97) and sex distribution was balanced (F:M ratio 149:153).

Basal cell carcinoma constituted the majority of the lesions (BCC; 74.2%, 224/302), followed by squamous cell carcinoma (SCC; 16.6%, 50/302), basosquamous carcinoma (BSC; 4.0%, 12/302), SCC in situ (4.6%, 14/302), basosquamous cell carcinoma (BSC; 3.0%, 10/336), and mixed BCC/SCC (<1%, 2/302). 

The most common anatomical location was the nose. Complete excision was documented in 72.1% (207/287) of cases with clear excision status. Characteristic of the study population are shown in Table 1.

In recurrence group comparisons, younger age was associated with recurrence—the median age in the recurrence group was 70 years (IQR 63–78) vs. 75 years in the non-recurrence group (IQR 67–81). Cancer type did not differ significantly between recurrence and non-recurrence groups (*p* = 0.1058), although SCC showed a higher recurrence rate than the overall cohort (38.78%, 19/49). Among BCC lesions with available subtype data, infiltrative variants demonstrated higher recurrence rates (27.10%, 29/107) compared with nodular (19.61%, 10/51) and superficial (13.64%, 3/22) subtypes.

Sex, smoking history, and immunosuppression were not associated with recurrence (all *p* > 0.05). Prior radiotherapy was associated with recurrence (58.33%, 7/12; *p* = 0.0418). Complete excision was not associated with recurrence (*p* = 0.2198), with recurrence observed in 31.58% (24/76) of incompletely excised lesions. Multiple-tumour status was associated with recurrence (*p* = 0.0164). Tumor dimension, excised specimen area, and smallest surgical margin width did not differ significantly between groups (*p* = 0.9458, *p* = 0.6866, and *p* = 0.4468, respectively).

Univariate logistic regression showed lower odds of recurrence with increasing age (OR 0.97 per year, 95% CI 0.950–0.993; *p* = 0.0110) and for age > 65 years (OR 0.41, 95% CI 0.226–0.737; *p* = 0.0028).

Prior radiotherapy was associated with higher odds of recurrence (OR 3.84, 95% CI 1.190–13.329; *p* = 0.0252). High-risk histology was not significantly associated with recurrence (OR 1.54, 95% CI 0.911–2.624; *p* = 0.1111). In a multivariable model restricted to BCC and SCC (n = 253), associations were attenuated (SCC vs. BCC OR 1.88, 95% CI 0.876–3.955; *p* = 0.0981; incomplete excision OR 1.62, 95% CI 0.866–2.982; *p* = 0.1265). Model discrimination was modest (AUC 0.61, 95% CI 0.54–0.69).

**Figure 1:**
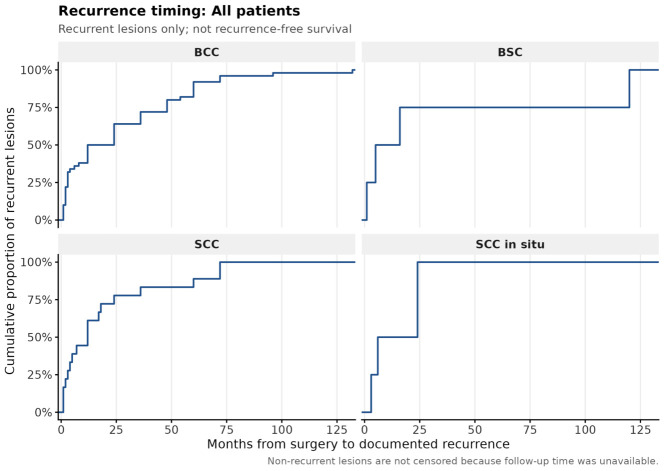
Cumulative distribution of time (months) from surgery to documented recurrence among recurrent lesions, stratified by diagnosis. The figure includes only lesions with documented recurrence and available timing data and does not represent a Kaplan–Meier recurrence-free survival estimate.

Among recurrent lesions with available timing data, time to recurrence varied across diagnostic groups. The median time to recurrence was 18.0 months for BCC (n = 50; IQR 3.0–48.0), 12.0 months for SCC (n = 18; IQR 3.2–22.5), and 15.0 months for SCC in situ (n = 4; IQR 5.2–24.0). For BSC (n = 4), the median time to recurrence was 10.5 months (IQR 4.0–42.0). Most recurrences occurred within the first two years following surgery, although late events were observed, with maximum times reaching up to 132 months for BCC and 72 months for SCC. The distribution of time to recurrence among recurrent lesions is presented in Figure 1.

**Figure 2:**
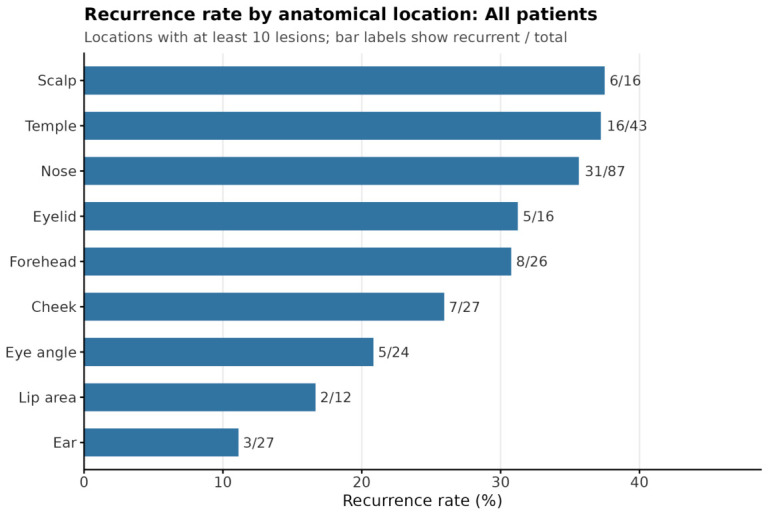
Recurrence rate by anatomical location. Bars represent the proportion of recurrent lesions for each site, with labels indicating recurrent/total counts. Only locations with at least 10 lesions are shown.

Recurrence rates varied by anatomical site, with the highest rate observed on the scalp (6/16, 37.50%), followed by the temple (16/43, 37.21%) and nose (31/87, 35.63%). Intermediate rates were seen on the eyelid (5/16, 31.25%) and forehead (8/26, 30.77%), while lower rates were observed on the ear (3/27, 11.11%). Missing data were substantial for tumor dimension (58/302) and smallest surgical margin (71/302). Recurrence was higher among lesions with missing tumor dimension data (42.11% vs. 25.21%; *p* = 0.0142). The association between tumor recurrence and analyzed variables are shown it Table 2. Recurrence rates by anatomical location are presented in Figure 2. 

## 4. Discussion

In this retrospective, single-center study, the recurrence patterns of surgically treated non-melanoma head and neck skin cancers were assessed. Risk factors associated with an increased risk of local recurrence have also been identified. The overall recurrence rate of 28.52% observed in the present study is higher than many previous studies, which typically report recurrence rates of less than 10% for BCC and up to 20% for SCC [1,15,17,19,20,21]. This difference may result from the inclusion of only patients with head and neck skin cancers, which are considered high-risk locations for recurrence. The high recurrence rate in this retrospective is directly correlated with the center’s reference status. High-reference centers treat advanced lesions that are anatomically more challenging, therefore more difficult to treat with complete excision and predisposing to possible recurrence. Patients with less demanding cases of facial skin cancer, qualified for surgical excision, are referred to smaller centers, which likely accounts for the lower recurrence rate among patients at that center.

Basal cell carcinoma represented the largest group of cancers, whereas squamous cell carcinoma formed a smaller but more aggressive subgroup. This is consistent with epidemiological data indicating that BCC accounts for approximately 70–80% of non-melanoma skin cancers [8,11,22,23,24]. SCC demonstrated a higher recurrence rate than the general population, in accordance with previous reports describing its increased local invasiveness and recurrence potential [16,25,26,27].

Among the subtypes, in our cohort infiltrative (morpheaform) BCC subtypes exhibited higher recurrence rates compared with nodular and superficial variants. This matched previous findings suggesting that aggressive histological growth patterns strongly predicted recurrence [28,29,30,31,32].

The risk of recurrence was strongly correlated with tumor location. The highest recurrence rate was observed for lesions located on the scalp, temple and nose, followed by tumors of the eyelids, and forehead. These locations significantly overlap with the facial H-zone, which is commonly described as an area of increased cancer risk due to embryonic fusion planes, thin or variable dermis, and proximity to critical anatomical structures [14,33,34,35,36]. Structural integrity within the H-zone-nasal, periorbital and auricular regions remains crucial for both functional and aesthetic causes. In these anatomically challenging areas, wider surgical margins may substantially increase wound tension, thereby rendering closure and reconstruction more difficult. This often limits the extent of excision that can be safely achieved. As a result, maintaining an adequate margin in these locations can be limited. Combined with the technical difficulty of the surgical procedure, it may increase the likelihood of incomplete excision and therefore microscopic residual disease, thereby contributing to increased risk of cancer recurrence [16,37,38]. For functional and aesthetic reasons, maintaining an adequate margin in these locations can be limited. This, combined with the technical difficulty of the surgical procedure, may increase the risk of microscopic residual disease and lead to the cancer recurrence [16,37,38].

Interestingly, despite older age being a well-established risk factor for skin cancer development, a higher recurrence rate was observed in younger individuals. This is supported by previous studies showing more aggressive tumor behavior and higher recurrence rates in younger patients. Such findings may reflect underlying biological differences, genetic predisposition, or cumulative patterns of ultraviolet radiation exposure [33,39,40,41]. Regarding older patients, cancer is primarily correlated with cumulative exposure to UV radiation for many years. Younger people have less exposure to UV radiation, so the occurrence of skin cancers at an early age may be related to a genetic predisposition. Therefore, younger patients are more likely to have a more aggressive course of cancer, and these patients should be monitored closely, as the onset of cancer at a younger age predisposes them to developing further cancers due to genetic factors.

Prior radiotherapy was a significant predictor of recurrence. This correlation is aligned with previous reports demonstrating that irradiated skin shows impaired healing capacity, dysfunctional immune surveillance, and an increased tendency for aggressive tumor behavior and radioresistance [3,42,43,44]. These reports emphasize the importance of careful follow-up of patients with a history of skin radiotherapy.

Notably, tumor size, surgical margin width, and completeness of excision were not significantly associated with recurrence, despite the fact that incompletely excised tumors showed a numerical trend toward higher recurrence. These observations were consistent with those reported in other retrospective studies, suggesting that margin status alone may not be sufficient to predict recurrence in high-risk facial tumors [5,6,45,46]. However, in cases where data on tumor dimensions and margins were missing, the recurrence rate was substantially higher (42.11% vs. 25.21%; *p* = 0.0142). This likely reflects an overrepresentation of the most complex and difficult cases within the subgroup with missing data, particularly in lesions for which histopathological reports focused on the assessment of surgical radicality rather than exact measurements. Therefore, the prognostic value of tumor dimensions and surgical margins in our statistical models may be underestimated as high-risk recurrent cases were insufficiently represented in the evaluated variables. Consequently, the lack of statistical significance for these variables in our model should be interpreted with caution, and their potential predictive role as risk factors for recurrence should remain clinically relevant.

Considering that multivariate models based solely on clinical and histopathological variables showed only moderate discriminatory ability in this and in previous studies, it can be concluded that additional factors, i.e., surgeon experience, reconstruction method, molecular characteristics of the tumor, and patient compliance, also contribute to the increased risk of recurrence [8,47,48,49,50].

Diagnosis-specific median times to recurrence ranged from 10.5 to 18 months, with most events occurring within the first two years following surgery; this pattern is consistent with the literature and supports the need for close, early surveillance of patients with a history of skin cancer [11,35,36,51].

### Limitations

Retrospective design: Subject to selection bias, information bias, and unmeasured confounding. Associations should not be interpreted causally.

Missing data not at random: Higher recurrence rate when tumor dimension is missing (42.11% vs. 25.21% (*p* = 0.0142). These missing measurements likely relate to the most difficult cases, where reporting was less standardized. As a result, the predictive value of tumor size and margins may be underestimated in our analysis.

No external validation: Findings are specific to this institution and patient population. External validation in independent cohorts is essential before clinical application.

Confounding by indication: Radiotherapy and “multiple tumors” associations likely reflect treatment selection rather than causal effects. These variables should not be used to guide clinical decisions without accounting for confounding.

Time-to-event analysis limitation: Only recurrent lesions contributed time data; follow-up time for non-recurrent lesions was not available, precluding recurrence-free survival analysis with censoring. The analysis therefore reflects the distribution of time to recurrence among events rather than a Kaplan–Meier estimate.

Outcome definition: “Recurrence” was defined based on histopathological confirmation of the same tumor type occurring at the identical anatomical site after excision; however, despite this definition, it cannot be completely excluded that some cases classified as recurrence may in fact represent new primary tumors arising within a field of cancerization in the same region. Nevertheless, such situations are considered extremely rare and are unlikely to have meaningfully influenced the results of the present study.

## 5. Conclusions

Recurrence of non-melanoma skin cancers in the head and neck region remains frequent in clinical practice, especially in high-risk anatomical locations and aggressive histological subtypes. These findings highlight the importance of long-term follow-up and support further prospective studies to improve recurrence risk assessment and treatment strategies. Due to the high risk of recurrence of skin cancers on the head and neck area and their more aggressive course, it should be remembered that such patients require intensified screening to detect possible recurrence at an early stage. It is important to remember to also include people who had previously undergone radiotherapy in this follow-up.

## Figures and Tables

**Table 1:** Table highlighting the characteristics of the study population.

Study Population Summary, All Patients		
Parameter	Value	
Eligible lesions (NMSC and SCC in situ)	302	
Patients contributing eligible lesions	132	
Lesions with known recurrence outcome	291	
Recurrent lesions	83	
Recurrence rate	28.52% (83/291)	
Median age, years (IQR)	73.0 (66.0–81.0)	
Female:Male (lesions)	149:153	
Most common diagnosis	BCC	
Most common anatomical location	Nose	
Baseline Characteristics, Categorical, All Patients		
Variable	Level	N (%)
Sex	Female	149 (49.3%)
	Male	153 (50.7%)
Diagnosis	BCC	224 (74.2%)
	BSC	12 (4.0%)
	Mixed BCC/SCC	2 (0.7%)
	SCC	50 (16.6%)
	SCC in situ	14 (4.6%)
Location risk group	Standard-risk	84 (27.9%)
	High-risk	217 (72.1%)
Complete excision	No	80 (27.9%)
	Yes	207 (72.1%)
Nerve or vessel invasion	No	287 (97.6%)
	Yes	7 (2.4%)
Metastases	No	294 (99.7%)
	Yes	1 (0.3%)
Multiple tumors	No	200 (67.3%)
	Yes	97 (32.7%)
History of skin cancer	No	121 (40.7%)
	Yes	176 (59.3%)
Smoking	No	211 (69.9%)
	Yes	91 (30.1%)
Prior radiotherapy	No	286 (95.7%)
	Yes	13 (4.3%)
Immunosuppression	No	279 (93.6%)
	Yes	19 (6.4%)
Age group	≤50	12 (4.0%)
	51–65	52 (17.3%)
	66–75	104 (34.6%)
	>75	133 (44.2%)

**Table 2:** Table showing the association between tumor recurrence and analyzed variables.

Variable	Level	Total Lesions at Level	Recurrent	Non-Recurrent	Recurrence Rate (%)	Cramer’s V	*p* Value
Sex	Female	144	41	103	28.47	0.001	10.000
	Male	147	42	105	28.57		
Anatomical location	Nose	87	31	56	35.63	0.227	0.1456
	Temple	43	16	27	37.21		
	Cheek	27	7	20	25.93		
	Ear	27	3	24	11.11		
	Forehead	26	8	18	30.77		
	Eye angle	24	5	19	20.83		
	Eyelid	16	5	11	31.25		
	Scalp	16	6	10	37.50		
	Lip area	12	2	10	16.67		
	Chin/Jaw	8	0	8	0.00		
	Neck	4	0	4	0.00		
Location risk group	Standard-risk	81	21	60	25.93	0.037	0.5652
	High-risk	209	62	147	29.67		
Diagnosis	BCC	216	54	162	25.00	0.150	0.1058
	SCC	49	19	30	38.78		
	SCC in situ	14	4	10	28.57		
	BSC	10	5	5	50.00		
	Mixed BCC/SCC	2	1	1	50.00		
Complete excision	No	76	24	52	31.58	0.078	0.2198
	Yes	201	48	153	23.88		
Nerve or vessel invasion	No	277	74	203	26.71	0.106	0.0929
	Yes	7	4	3	57.14		
Metastases	No	284	77	207	27.11	0.097	0.2737
	Yes	1	1	0	100.00		
Multiple tumors	No	195	63	132	32.31	0.144	0.0164
	Yes	92	17	75	18.48		
History of skin cancer	No	118	32	86	27.12	0.014	0.8937
	Yes	169	48	121	28.40		
Smoking	No	203	55	148	27.09	0.048	0.4799
	Yes	88	28	60	31.82		
Prior radiotherapy	No	277	74	203	26.71	0.140	0.0418
	Yes	12	7	5	58.33		
Immunosuppression	No	270	77	193	28.52	0.041	0.6034
	Yes	19	4	15	21.05		
Age group	≤50	12	4	8	33.33	0.198	0.0100
	51–65	49	23	26	46.94		
	66–75	99	28	71	28.28		
	>75	130	28	102	21.54		
Smallest margin category	<1 mm	53	16	37	30.19	0.088	0.6589
	1-<2 mm	61	15	46	24.59		
	2-<5 mm	89	25	64	28.09		
	≥5 mm	19	3	16	15.79		

Fisher’s exact test. Cramér’s V = effect size (0.1 small, 0.3 medium, 0.5 large).

## Data Availability

The data underlying this article cannot be shared publicly due to the privacy of individuals that participated in the study. The data will be shared on reasonable request to the corresponding author.
